# Associations of eating a late-evening meal before bedtime with low serum amylase and unhealthy conditions

**DOI:** 10.1186/2251-6581-12-53

**Published:** 2013-12-19

**Authors:** Haruki Oshida, Ayano Kutsuma, Kei Nakajima

**Affiliations:** Division of Clinical Nutrition, Department of Medical Dietetics, Faculty of Pharmaceutical Sciences, Josai University, 1-1 Keyakidai, Sakado, Saitama, 350-0295 Japan

**Keywords:** Late-evening meal, Low serum amylase, Skipping breakfast, Eating behaviors

## Abstract

Little is known about the associations of eating a late-evening meal (ELM), a putative unhealthy eating behavior, with low serum amylase, other eating behaviors, and cardiometabolic risk factors. Therefore, we investigated whether ELM before bedtime was associated with low serum amylase or other clinical factors in 2,426 asymptomatic adults aged 20–80 years. Multivariate logistic regression analysis showed that ELM was significantly associated with low serum amylase (<60 IU/l), overweight, smoking, daily alcohol consumption, skipping breakfast, and rapid eating, but not with abnormal glucose metabolism. In conclusion, ELM may be independently associated with low serum amylase and common unhealthy behaviors.

## Findings

Although eating a late-evening meal (ELM) is likely to be an unhealthy behavior that may cause postprandial hyperglycemia and decrease the metabolic rate[1–3], the physiological and clinical relevance of ELM is poorly understood. In our previous studies, low serum amylase was associated with obesity, type 2 diabetes, metabolic syndrome, and non-alcoholic fatty liver disease[4, 5]. Therefore, we investigated whether ELM was associated with low serum amylase and other unhealthy conditions or eating behaviors in 2,426 asymptomatic adults.

The subjects were asymptomatic adults aged 20–80 years who lived in Saitama, Japan, and underwent thorough medical checkups at Social Insurance Omiya General Hospital, Saitama, Japan. This study was conducted using data collected between 2009 and 2010. The protocol was approved by the Ethics Committee of Josai University, and informed consent was obtained from all participants. Subjects with suspected acute pancreatitis (serum amylase >200 IU/l), very low serum amylase (<30 IU/l), or suspected renal failure were excluded from the study. Subjects completed a questionnaire, which was developed by the Japanese Ministry of Health, Labour and Welfare in 2007 to prevent metabolic syndrome and cardiovascular diseases[6]. Suspected ELM, skipping breakfast, and rapid eating were determined using the questions, “Do you eat a late-evening meal within 2 hour before bedtime at least three times per week?”, “Do you skip breakfast at least three times per week?”, and “Do you think your meal-eating speed is usually rapid compared with other people’s?”, respectively, for which the available responses were yes or no.

Anthropometric and laboratory tests were conducted after an overnight fast. Serum parameters were measured using an autoanalyzer (Hitachi, Tokyo, Japan). Serum amylase levels were measured using an enzymatic method (L-type Amylase; Wako, Tokyo, Japan) with a normal range of 41–112 IU/l. We defined low serum amylase as <60 IU/l, as previously described[5]. HbA1c was converted to National Glycohemoglobin Standardization Program levels[7].

Table 1 shows the clinical characteristics of the subjects. Overall, subjects who reported ELM were younger, which likely affected the less proportion of dyslipidemia, more frequently males, smokers, and daily alcohol consumers, had a higher body mass index, and frequently reported unhealthy eating behaviors. Multivariate logistic regression analysis showed that ELM was independently associated with low serum amylase and common unhealthy behaviors (Table 2, Model 4). However, elevated fasting plasma glucose and HbA1c (≥6.5%) were not significantly associated with ELM. These latter results suggest that ELM is not directly associated with impaired glucose metabolism, even though plasma glucose levels were transiently increased after a single late-evening meal and subsequent breakfast[8]. When the associations of low serum amylase with self-reported skipping breakfast and rapid eating were examined, no significant association was observed after adjustment for confounders listed in Table 2 Model 4 [odds ratio (95% CI), 1.20 (0.94-1.54), P = 0.15 and 0.99 (0.81-1.22), P = 0.96, respectively].

**Table 1:** **Subject characteristics**

	ELM		P value
	Absent	Present	
N	1,621	805	
Men, (%)	56.8	82.6	< 0.0001
Age	56.0 ± 11.8	48.8 ± 10.7	< 0.0001
BMI (kg/m^2^)	22.9 ± 3.1	24.0 ± 3.4	< 0.0001
Systolic blood pressure (mmHg)	122 ± 18.8	120 ± 18.9	0.18
Diastolic blood pressure (mmHg)	75.4 ± 12.5	76.1 ± 13.1	0.16
Triglyceride (mg/dl)	91 (66–133)	101 (71–144)	< 0.0001
HDL-cholesterol (mg/dl)	62.5 ± 14.8	57.9 ± 14.6	< 0.0001
FPG (mg/dl)	101 ± 18.5	102 ± 17.2	0.30
HbA1c (%, NGSP)	5.7 ± 0.6	5.6 ± 0.6	0.0005
eGFR (ml/min/1.73 m^2^)	75.2 ± 14.0	78.3 ± 13.1	< 0.0001
Serum amylase (IU/l)	76.2 ± 23.8	69.2 ± 22.0	< 0.0001
Medications for			
Hypertension, n (%)	293 (18.1)	118 (14.7)	0.03
Diabetes, n (%)	60 (3.7)	21 (2.6)	0.16
Dyslipidemia, n (%)	205 (12.6)	47 (5.8)	< 0.0001
Past history			
Heart disease, n (%)	29 (1.8)	12 (1.5)	0.59
Stroke, n (%)	65 (4.0)	20 (2.5)	0.05
Current smoking, n (%)	318 (19.6)	311 (38.6)	< 0.0001
Daily alcohol consumption, n (%)	419 (25.8)	339 (42.1)	< 0.0001
Regular exercise, n (%)	605 (37.3)	166 (20.6)	< 0.0001
Self-reported skipping breakfast, n (%)	159 (9.8)	232 (28.8)	< 0.0001
Self-reported rapid eating, n (%)	406 (25.0)	313 (38.9)	< 0.0001

Data are expressed as means ± SD, median (interquartile range), or *n* (%).

Differences in continuous and categorical variables between the two groups were examined using *t*-tests and χ^2^-tests, respectively, except for triglyceride, which was compared using the Mann–Whitney test.

eGFR was calculated as previously described[5].

BMI, body mass index; eGFR, estimated glomerular filtration rate; ELM, eating a late-evening meal; FPG, fasting plasma glucose; HbA1c, hemoglobin A1c; HDL, high-density lipoprotein; NSGP, National Glycohemoglobin Standardization Program.

**Table 2:** **Odds ratios of low serum amylase and other clinical variables for ELM**

	OR (95% CI)	P value
Low serum amylase		
Model 1	1.86 (1.55–2.23)	<0.0001
Model 2	1.47 (1.20–1.79)	0.0002
Model 3	1.41 (1.16–1.73)	0.0008
Model 4	1.33 (1.08–1.63)	0.008
Men		
Model 1	3.61 (2.94–4.44)	<0.0001
Model 4	2.28 (1.80–2.90)	<0.0001
Current smoking		
Model 1	2.58 (2.14–3.11)	<0.0001
Model 4	1.28 (1.03–1.59)	0.03
Daily alcohol drinking		
Model 1	2.48 (2.01–3.05)	<0.0001
Model 4	1.83 (1.43–2.34)	<0.0001
Regular exercise		
Model 1	0.44 (0.36–0.53)	<0.0001
Model 4	0.66 (0.53–0.83)	0.0004
Overweight (BMI ≥25.0 kg/m^2^)		
Model 1	1.89 (1.57–2.29)	<0.0001
Model 4	1.38 (1.11–1.71)	0.004
Self–reported breakfast skipping		
Model 1	3.72 (2.98–4.66)	<0.0001
Model 4	2.18 (1.70–2.80)	<0.0001
Self–reported rapid eating		
Model 1	1.90 (1.59–2.28)	<0.0001
Model 4	1.60 (1.30–1.96)	<0.0001
Elevated FPG (≥126 mg/dl)		
Model 1	0.95 (0.67–1.36)	0.79
Model 4	0.91 (0.58–1.43)	0.62
Elevated HbA1c (≥6.5%)		
Model 1	0.96 (0.68–1.35)	0.82
Model 4*	1.13 (0.71–1.79)	0.61

Model 1: unadjusted.

Model 2: adjusted for age (as a continuous variable), sex, and current smoking (versus non-smoking).

Model 3: Model 2 plus adjustment for medications (for hypertension, diabetes, and dyslipidemia), history of stroke or cardiovascular disease, daily alcohol consumption (versus infrequent/no alcohol consumption), and regular exercise (≥30 min exercise per session >2 times/week versus infrequent exercise).

Model 4: Model 3 plus overweight (BMI ≥25.0 kg/m^2^; versus non-overweight, BMI <25 kg/m^2^), self-reported skipping breakfast (yes versus no), self-reported rapid eating (versus normal or slow), eGFR (as a continuous variable), and elevated FPG (≥126 mg/dl; versus normal FPG, <126 mg/dl).

*Elevated FPG was not adjusted.

eGFR, estimated glomerular filtration rate; FPG, fasting plasma glucose; HbA1c, hemoglobin A1c.

In recent years, unfavorable eating behaviors, such as rapid eating and skipping breakfast, were reported to be associated with obesity and abnormal glucose metabolism[9–14]. The serum amylase level is proportional to the serum level of pancreatic amylase that leaks from pancreas into the blood[15, 16]. Because pancreatic amylase plays a key role in digestion, the serum amylase level reflects the state of digestion, particularly of carbohydrate. Theoretically, low levels of secreted amylase represent reduced absorption of carbohydrate, the predominant energy source in most meals, and may result in malnutrition and underweight. Unexpectedly, in our previous studies of asymptomatic adults, low serum amylase was associated with obesity and metabolic syndrome[4], which commonly reflect excess energy intake. Additionally, low levels of salivary amylase, which also contributes to the digestion of food[17], were observed in diabetic patients[18]. Hyperglycemia after a high-starch load occurred in healthy subjects with low salivary amylase[19]. Therefore, the associations among ELM, low serum amylase, and overweight are not inconsistent with these previous findings. A plausible explanation for the association is that low serum amylase may reflect an adaptive process to protect against prolonged hyperglycemia with regular ELMs and overweight.

Our study has several limitations. First, the quantity, type, and precise timing of the evening meal were not recorded. Second, the questionnaire used to assess eating behaviors has not been validated. However, the significant associations of ELM with other abnormal eating behaviors, particularly skipping breakfast and rapid eating, indicate that the simple question about ELM may be sufficient to suspect ELM and other abnormal eating behaviors. Nevertheless, the limited data and the design of the questionnaire could interfere with the observed associations. Third, other digestive enzymes such as lipase and trypsin were not measured in this study. Therefore, further studies are needed to confirm the current findings and to clarify the underlying mechanism and the causality in more detail.

In conclusion, the current results suggest that ELM is independently associated with low serum amylase and common unhealthy behaviors.

## Abbreviations

ELM: Eating a late-evening meal.

## References

[1] Frank SA, Roland DC, Sturis J, Byrne MM, Refetoff S, Polonsky KS, Van Cauter E (1995). Effects of aging on glucose regulation during wakefulness and sleep. Am J Physiol.

[2] Katayose Y, Tasaki M, Ogata H, Nakata Y, Tokuyama K, Satoh M (2009). Metabolic rate and fuel utilization during sleep assessed by whole-body indirect calorimetry. Metabolism.

[3] Morse SA, Ciechanowski PS, Katon WJ, Hirsch IB (2006). Isn't this just bedtime snacking? The potential adverse effects of night-eating symptoms on treatment adherence and outcomes in patients with diabetes. Diabetes Care.

[4] Nakajima K, Nemoto T, Muneyuki T, Kakei M, Fuchigami H, Munakata H (2011). Low serum amylase in association with metabolic syndrome and diabetes: a community-based study. Cardiovasc Diabetol.

[5] Nakajima K, Oshida H, Muneyuki T, Saito M, Hori Y, Fuchigami H, Kakei M, Munakata H (2013). Independent association between low serum amylase and non-alcoholic fatty liver disease in asymptomatic adults: a cross-sectional observational study. BMJ Open.

[6] Ministry of Health, Labour, and Welfare (2008). Health Examination and Guidance Program for Japanese Adults.

[7] Kashiwagi A, Kasuga M, Araki E (2012). International clinical harmonization of glycated hemoglobin in Japan: from Japan Diabetes Society to National Glycohemoglobin Standardization Program values. J Diabetes Investig.

[8] Sato M, Nakamura K, Ogata H, Miyashita A, Nagasaka S, Omi N, Yamaguchi S, Hibi M, Umeda T, Nakaji S, Tokuyama K (2011). Acute effect of late evening meal on diurnal variation of blood glucose and energy metabolism. Diabetes Res Clin Pract.

[9] Leong SL, Madden C, Gray A, Waters D, Horwath C (2011). Faster self-reported speed of eating is related to higher body mass index in a nationwide survey of middle-aged women. J Am Diet Assoc.

[10] Sakurai M, Nakamura K, Miura K, Takamura T, Yoshita K, Nagasawa SY, Morikawa Y, Ishizaki M, Kido T, Naruse Y, Suwazono Y, Sasaki S, Nakagawa H (2012). Self-reported speed of eating and 7-year risk of type 2 diabetes mellitus in middle-aged Japanese men. Metabolism.

[11] Ohkuma T, Fujii H, Iwase M, Kikuchi Y, Ogata S, Idewaki Y, Ide H, Doi Y, Hirakawa Y, Mukai N, Ninomiya T, Uchida K, Nakamura U, Sasaki S, Kiyohara Y, Kitazono T (2013). Impact of eating rate on obesity and cardiovascular risk factors according to glucose tolerance status: the Fukuoka Diabetes Registry and the Hisayama Study. Diabetologia.

[12] Timlin MT, Pereira MA (2007). Breakfast frequency and quality in the etiology of adult obesity and chronic diseases. Nutr Rev.

[13] Smith KJ, Gall SL, McNaughton SA, Blizzard L, Dwyer T, Venn AJ (2010). Skipping breakfast: longitudinal associations with cardiometabolic risk factors in the childhood determinants of adult health study. Am J Clin Nutr.

[14] Deshmukh-Taskar P, Nicklas TA, Radcliffe JD, O'Neil CE, Liu Y (2012). The relationship of breakfast skipping and type of breakfast consumed with overweight/obesity, abdominal obesity, other cardiometabolic risk factors and the metabolic syndrome in young adults. The National Health and Nutrition Examination Survey (NHANES): 1999-2006. Public Health Nutr.

[15] Skrha J, Stĕpán J (1987). Clinical significance of amylase isoenzyme determination. Acta Univ Carol Med Monogr.

[16] Levitt MD, Ellis CJ, Murphy SM, Schwartz ML (1981). Study of the possible enteropancreatic circulation of pancreatic amylase in the dog. Am J Physiol.

[17] Santos JL, Saus E, Smalley SV, Cataldo LR, Alberti G, Parada J, Gratacòs M, Estivill X (2012). Copy number polymorphism of the salivary amylase gene: implications in human nutrition research. J Nutrigenet Nutrigenomics.

[18] Panchbhai AS, Degwekar SS, Bhowte RR (2010). Estimation of salivary glucose, salivary amylase, salivary total protein and salivary flow rate in diabetics in India. J Oral Sci.

[19] Mandel AL, Breslin PA (2012). High endogenous salivary amylase activity is associated with improved glycemic homeostasis following starch ingestion in adults. J Nutr.

